# Hepatic ferroptosis plays an important role as the trigger for initiating inflammation in nonalcoholic steatohepatitis

**DOI:** 10.1038/s41419-019-1678-y

**Published:** 2019-06-18

**Authors:** Shinya Tsurusaki, Yuichi Tsuchiya, Tomoko Koumura, Misaki Nakasone, Taro Sakamoto, Masaki Matsuoka, Hirotaka Imai, Cindy Yuet-Yin Kok, Hitoshi Okochi, Hiroyasu Nakano, Atsushi Miyajima, Minoru Tanaka

**Affiliations:** 1 Department of Regenerative Medicine, Research Institute, National Center for Global Health and Medicine, Tokyo, Japan; 2 Laboratory of Stem Cell Regulation, Institute for Quantitative Biosciences, The University of Tokyo, Tokyo, Japan; 3 Department of Biochemistry, Toho University School of Medicine, Tokyo, Japan; 4 School of Pharmaceutical Sciences, Kitasato University, Tokyo, Japan; 5 Laboratory of Stem Cell Therapy, Institute for Quantitative Biosciences, The University of Tokyo, Tokyo, Japan; 6 Present Address: Centre for Heart Research, The Westmead Institute for Medical Research, Westmead, NSW Australia

**Keywords:** Non-alcoholic steatohepatitis, Acute inflammation

## Abstract

Nonalcoholic steatohepatitis (NASH) is a metabolic liver disease that progresses from simple steatosis to the disease state of inflammation and fibrosis. Previous studies suggest that apoptosis and necroptosis may contribute to the pathogenesis of NASH, based on several murine models. However, the mechanisms underlying the transition of simple steatosis to steatohepatitis remain unclear, because it is difficult to identify when and where such cell deaths begin to occur in the pathophysiological process of NASH. In the present study, our aim is to investigate which type of cell death plays a role as the trigger for initiating inflammation in fatty liver. By establishing a simple method of discriminating between apoptosis and necrosis in the liver, we found that necrosis occurred prior to apoptosis at the onset of steatohepatitis in the choline-deficient, ethionine-supplemented (CDE) diet model. To further investigate what type of necrosis is involved in the initial necrotic cell death, we examined the effect of necroptosis and ferroptosis inhibition by administering inhibitors to wild-type mice in the CDE diet model. In addition, necroptosis was evaluated using mixed lineage kinase domain-like protein (MLKL) knockout mice, which is lacking in a terminal executor of necroptosis. Consequently, necroptosis inhibition failed to block the onset of necrotic cell death, while ferroptosis inhibition protected hepatocytes from necrotic death almost completely, and suppressed the subsequent infiltration of immune cells and inflammatory reaction. Furthermore, the amount of oxidized phosphatidylethanolamine, which is involved in ferroptosis pathway, was increased in the liver sample of the CDE diet-fed mice. These findings suggest that hepatic ferroptosis plays an important role as the trigger for initiating inflammation in steatohepatitis and may be a therapeutic target for preventing the onset of steatohepatitis.

## Introduction

Nonalcoholic fatty liver disease (NAFLD), a kind of lifestyle disease ranging from simple steatosis to steatohepatitis, is the most prevalent liver disease in the world. Nonalcoholic steatohepatitis (NASH) is a severe form of NAFLD that is characterized by lipid droplet accumulation in hepatocytes, hepatic cell death, infiltration of inflammatory cells, and mostly fibrosis. While the prognosis of simple steatosis is benign, the progression into NASH is a serious risk factor for cirrhosis and carcinogenesis^1,2^. Therefore, it is important to elucidate the mechanism underlying the pathological process from simple steatosis to steatohepatitis. Although the “two-hit theory” and “multiple parallel hit hypothesis” have been proposed in the pathogenesis of NASH, hepatic cell death triggered by abnormal lipid accumulation in hepatocytes is considered as a likely cause of inflammation^3^. Several studies have already reported that apoptosis and necroptosis are involved in the pathological process of NASH, by using cell death-related genetically modified mouse models or chemical inhibitors^4,5^. These studies have highlighted the contribution of each type of cell death to the progression of NASH based on relatively later symptoms such as fibrosis and carcinogenesis. However, the type of cell death involved in the onset of NASH has not been investigated, as it is quite difficult to specify the timing of cell death occurring after simple steatosis in long-term disease models. As such, the initial type of cell death that acts as the trigger for inflammation, and causes the onset of steatohepatitis from steatosis remains elusive. The aim of this study was to investigate the type of hepatocyte cell death involved in the onset of steatohepatitis. To focus on the earliest stage of hepatocyte cell death just before steatohepatitis, we explored apoptosis and necrotic cell death using the choline-deficient, ethionine-supplemented (CDE) diet model, which induces steatohepatitis in a relatively short period of time^6^. While the methionine-deficient, choline-deficient (MCD) or choline-deficient, l-amino acid-defined (CDAA) diet is a widely used mouse model of NASH^7–9^, the CDE model utilizes the supplementation of ethionine instead of methionine deficiency, which is a methionine analog for methionine starvation. We first determined the timeframe for the onset of steatohepatitis, based on elevation of serum markers for liver damage. We then aimed to identify the initial cell death that occurred within this timeframe. We have also established a method for distinguishing apoptosis and necrosis easily in the liver and have evaluated the type of hepatocyte cell death by using cell death-related inhibitory agents and knockout mice in this model. From these experiments, we discovered the role of ferroptosis as the initial cell death that triggers steatohepatitis.

Ferroptosis is a type of “programmed necrosis” recently identified as an iron- and lipid hydroperoxide-dependent nonapoptotic cell death in cancer cells. It has been reported that iron chelators as well as a few genes related to the lipid peroxide removal system such as cystine/glutamic acid transporter (xCT) and glutathione peroxidase 4 (GPx4) are involved in the suppression of ferroptosis^10,11^. It is also reported that hydroperoxidation of phosphatidylethanolamine (PE) within the lipid membrane causes induction of ferroptosis^12,13^. Currently, there is accumulating evidence that ferroptosis is involved in not only cell death of a certain type of tumor cells but also in the pathogenesis of several diseases such as neuronal dysfunction and acute kidney injury^14,15^. However, the role of ferroptosis in liver disease remains poorly understood.

In this study, we focus on the type of cell death that initially occurs at the earliest stage of NASH. Our data reveal that ferroptosis is the type of necrosis that precedes the other type of cell death, thus giving cues to initiate inflammation in this model. Our findings suggest the role of ferroptosis as a trigger at the onset of steatohepatitis.

## Results

### Pathological feature of liver injury after the CDE diet feeding

**Fig. 1:**
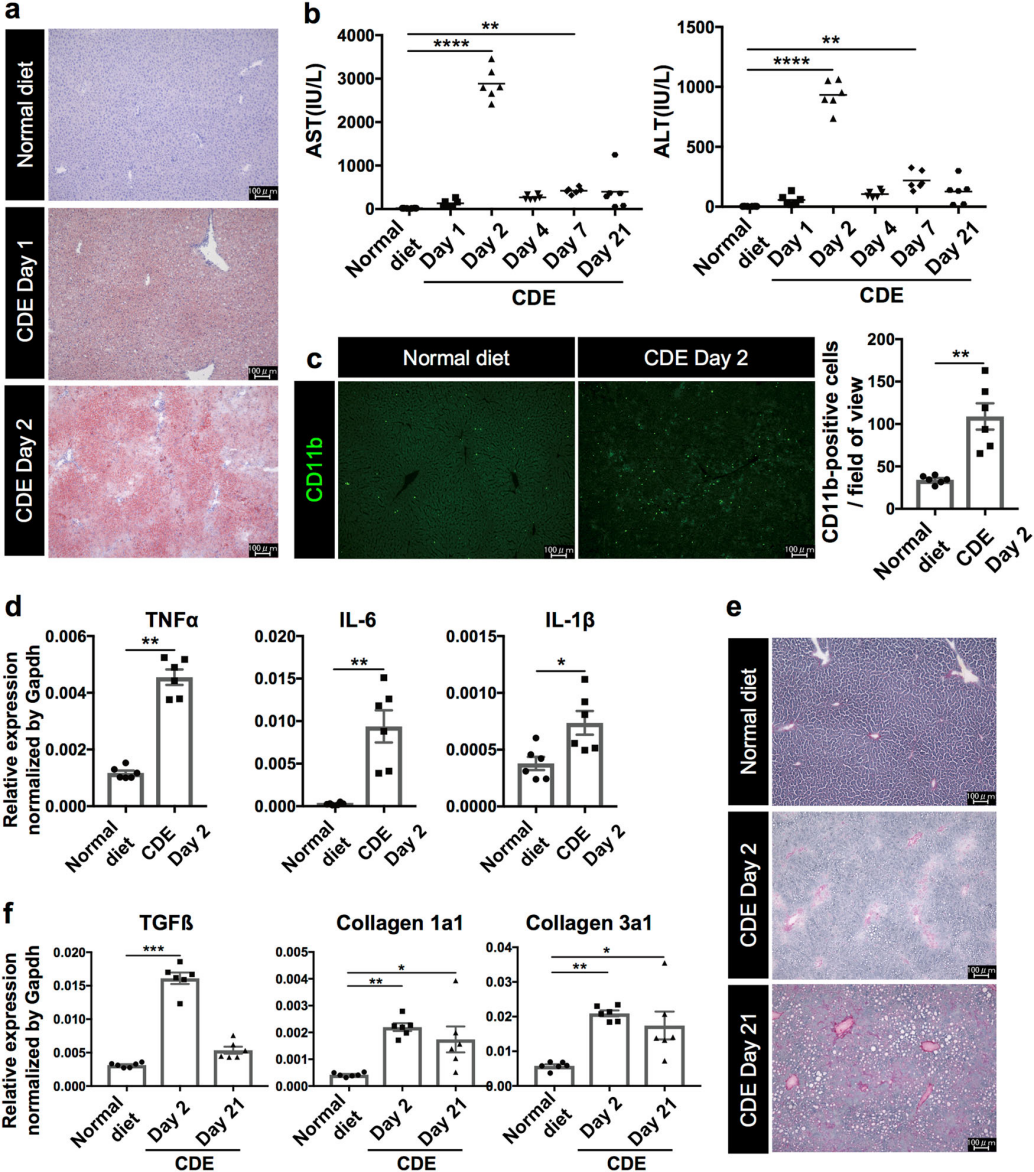
Pathological features of steatohepatitis in the CDE diet model. The lipid accumulation, inflammatory responses, and fibrosis in the liver were evaluated after choline-deficient, ethionine-supplemented (CDE) diet feeding. Six mice were used for analyses at each time point. **a** Representative images of lipid accumulation in the liver by Oil Red O staining after 0, 1, and 2 days post CDE diet feeding. **b** Measurement of serum liver injury markers, aspartate aminotransferase (AST) and alanine aminotransferase (ALT) after the CDE feeding (*n* = 6; **P *<* 0.01, ****P *<* 0.0001). **c** Fluorescence images of infiltration of immune cells by immunohistochemistry of liver sections for CD11b. The number of CD11b-positive cells was evaluated in 20 non-overlapping fields of view for each biological sample. The data are shown as the mean ± SEM (*n* = 6; **P *<* 0.01). **d** Gene expression analysis of inflammatory cytokines by quantitative reverse transcriptase PCR (RT-PCR). All data are normalized to Gapdh and shown as the means ± SEM (*n* = 6, *P *<* 0.05, **P *<* 0.01). **e** Representative images of liver fibrosis by Picro-Sirius Red staining. **f** Expression analysis of fibrosis-related genes by quantitative RT-PCR. All data are normalized to Gapdh and shown as the means ± SEM (*n* = 6; *P *<* 0.05, **P *<* 0.01, ***P < 0.001). Scale bar = 100 µm

The CDE diet is widely used as a murine chronic liver injury model that induces steatohepatitis and expansion of liver progenitor cells^16^. To characterize the pathological features of this model, we first investigated the status of NASH symptoms, i.e. fat accumulation, hepatocyte cell death, infiltration of immune cells, and fibrosis after the CDE diet feeding. The Oil Red O staining revealed that the increased fat deposition in hepatocytes was evident 1 day after CDE diet feeding and that drastic accumulation of lipid droplets was observed by 2 days after feeding (Fig. 1a). Next, we measured serum liver injury markers, aspartate aminotransferase (AST) and alanine aminotransferase (ALT) at several time points after the CDE diet feeding. Because hepatocytes are abundant in AST and ALT, the release of these two enzymes into serum represents the degree of hepatocyte cell death. As shown in Fig. 1b, the levels of both AST and ALT sharply increased after 2 days of the CDE feeding as previously reported^6^. Such a sharp increase of AST and ALT values was not detected after the CDAA-based high-fat diet feeding (Supplementary Fig. S1)^17^. In addition, infiltration of inflammatory cells was observed in the liver by immunostaining of CD11b, and gene expression of inflammatory cytokines was also upregulated (Fig. 1c, d). Thereafter, the levels of AST and ALT were decreased and maintained at a constant level, shifting to chronic liver injury phase. Consistent with the outcome of chronic hepatitis, the liver exhibited fibrosis at day 21 as evaluated by Picro-Sirius Red staining and expression analysis of fibrosis-related genes (Fig. 1e, f). Taken together, these results suggest that an initial cell death which triggers inflammation occurs within 2 days after CDE diet feeding in this model. Therefore, we further investigated the type of cell death at an early stage of steatohepatitis.

### Evaluation of cell death in the liver by establishment of in vivo necrosis detection method

**Fig. 2:**
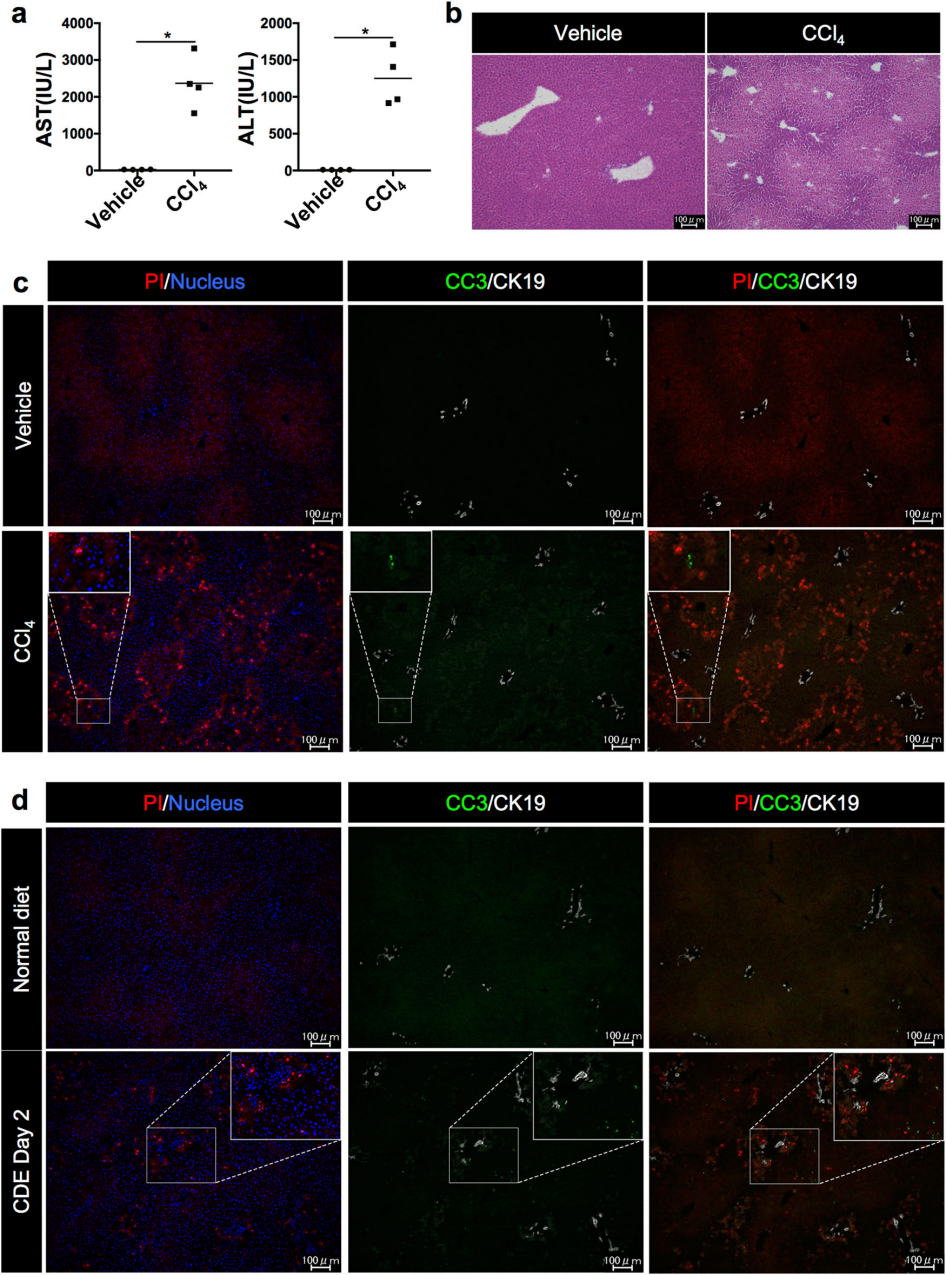
Evaluation of apoptotic and necrotic cells in the liver by immunohistochemistry for CC3 and in vivo necrosis assay. **a** Measurement of serum liver injury markers after 24 h of control (vehicle) and CCl_4_ treatment (*n* = 4; *P *<* 0.05). **b** HE staining of sections from control and CCl_4_-treated livers. **c** Fluorescence images of PI- and CC3-stained cells in control and CCl_4_-treated livers. **d** Fluorescence images of PI- and CC3-stained cells in the liver after 2 days of normal diet or the CDE diet feeding. Scale bar = 100 µm. PI propidium iodide, CCl_4_ carbon tetrachloride, CC3 cleaved caspase-3

While there are several methods for detecting apoptotic cells such as TUNEL, detection of single-strand DNA or activation of caspases, there is currently no method for convenient identification of necrosis in vivo. Electron microscopy is an excellent tool for distinguishing between apoptosis and necrosis; however, it is not suitable for observing several types of cell death simultaneously over a broad range of injured tissue. To establish a simple method to detect necrotic cells, we adapted the method of propidium iodide (PI) staining for in vivo study, which is widely used in vitro to identify necrosis. In necrotic cells, the collapsed plasma membrane allows PI to stain the cell nucleus, whereas it is excluded from early apoptotic cells with intact plasma membrane. To test whether this principle works well in vivo, we injected PI intravenously into carbon tetrachloride (CCl_4_)-treated mouse, which is a typical model of necrotic liver injury^18^. The liver damage induced by CCl_4_-treatment was confirmed by elevation of serum AST and ALT levels, and hematoxylin-eosin (HE) staining showed extensive necrotic areas around the central veins (CV) (Fig. 2a, b). PI staining revealed that numerous PI-positive cells were observed around CV in this model, whereas apoptotic cells were rarely detected by immunostaining of cleaved caspase-3 (CC3) (Fig. 2c). Co-staining of PI and CC3 demonstrated that most CC3-positive apoptotic cells are negative for PI (Fig. 2c). These results demonstrated that this in vivo necrosis assay can be used to detect necrotic cells. It was also possible to distinguish between apoptosis and necrosis by co-staining of PI with apoptosis-related marker in the early stages of hepatic cell death.

To identify the type of cell death at the onset of steatohepatitis, we applied the PI and CC3 staining method to the injured liver in the CDE model. Consistent with the rapid increase and subsequent decrease of serum AST and ALT (Fig. 1b) after CDE feeding, many PI-positive cells were detected in the injured liver after 2 days of CDE feeding, while a small number of PI-positive cells were observed 3 weeks later (Fig. 2d and Supplementary Fig. S2A). In contrast, no PI-positive cells were visible in normal liver (Fig. 2d). Immunostaining of CC3 revealed that several apoptotic cells were also detected in the same liver. However, CC3 and PI double-positive cells were hardly detected (Fig. 2d), suggesting that CC3-positive apoptotic cells and PI-positive necrotic cells existed independently in the injured liver. More intriguingly, we noticed that the initial cell death at day 2 seemed to occur predominantly at the portal region where is surrounding the portal vein and CK19-stained bile ducts (Fig. 2d). To further clarify the regional property in hepatic cell death, we compared the ratio of PI-positive cells between Cadherin1 (CDH1)-positive and -negative areas in the liver, because CDH1 is known to be preferentially expressed in hepatocytes at the portal region^19^. As a result, CDH1-positive region showed significantly higher ratio than CDH1-negative region (Supplementary Fig. S2B), suggesting that hepatic necrosis initially begins around the portal vein after CDE feeding.

### Necrosis precedes apoptosis at the onset of steatohepatitis

**Fig. 3:**
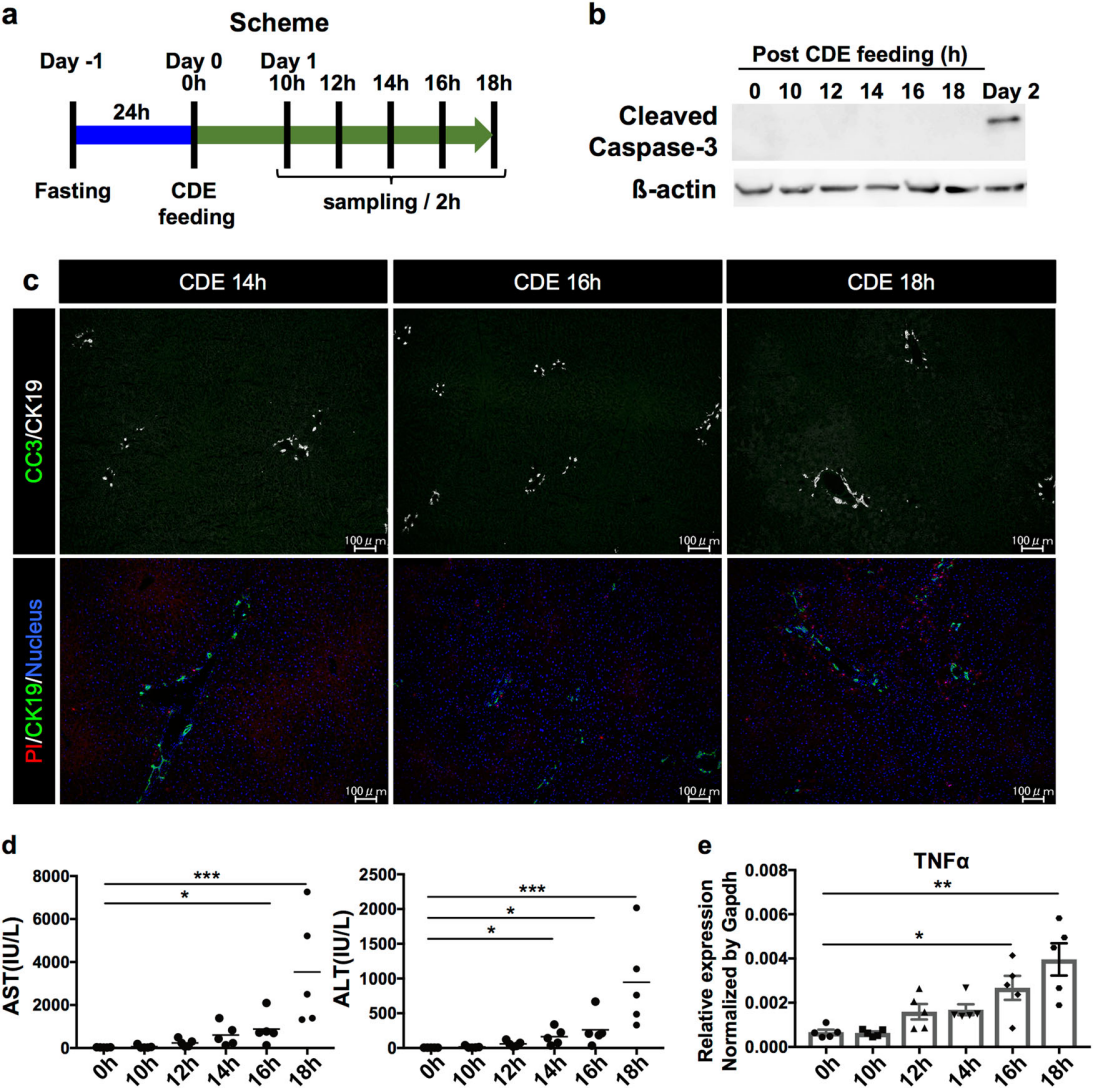
Detection of apoptosis and necrosis at an early stage of steatohepatitis in the CDE diet model. **a** Experimental design for identifying apoptosis and necrosis at the onset of steatohepatitis. **b** Western blot analysis of CC3 protein in the liver extracts after the CDE diet feeding. ß-Actin was used as an internal control. **c** Detection of apoptotic and necrotic cells in the liver after 14 to 18 h post CDE diet feeding by immunohistochemistry for CC3 and the in vivo necrosis assay. In images of apoptosis detection, CK19 is shown as white pseudocolor. **d** Measurement of serum liver injury markers after CDE diet feeding. (*n* = 5; *P *<* 0.05, ***P < 0.001). **e** Gene expression analysis of TNFα after the CDE diet feeding by quantitative RT-PCR. All data are normalized to Gapdh and shown as the mean ± SEM (*n* = 5; *P *<* 0.05, **P *<* 0.01). Scale bar = 100 µm

Since both apoptosis and necrosis had already occurred by day 2, we next investigated the status of fatty liver at an earlier time point to determine which cell death precedes the onset of steatohepatitis. To this end, we designed a new experimental scheme as shown in Fig. 3a. To standardize the time at which feeding commenced, mice were fasted for 24 h prior to feeding with CDE diet. After CDE diet was commenced (0 h), liver samples were analyzed at 10, 12, 14, 16, and 18 h post feeding. Unlike the injured liver at day 2, apoptosis could not be detected in the injured liver by either western blotting or CC3 immunostaining from 10 to 18 h post CDE diet feeding (Fig. 3b, c). In contrast, necrosis was observed as early as 14 h post feeding and thereafter the number of necrotic cell gradually increased (Fig. 3c and Supplementary Figure S3A, B). The PI-positive necrotic cells seemed to be mainly derived from CDH1-positive hepatocytes, although a minor contribution of CD11b-positive immune cells to necrosis was observed at 18 h post CDE diet feeding (Supplementary Fig. S3C, d). Consistent with this observation, the levels of serum liver injury markers and expression of tumor necrosis factor-α (TNFα) mRNA began to increase between 12 and 14 h after the CDE diet feeding (Fig. 3d, e). These results indicated that necrosis occurred prior to apoptosis, and is thereafter likely to be the trigger for the onset of steatohepatitis in this model.

### Necroptosis is not the cell death that initiates steatohepatitis in the CDE model

**Fig. 4:**
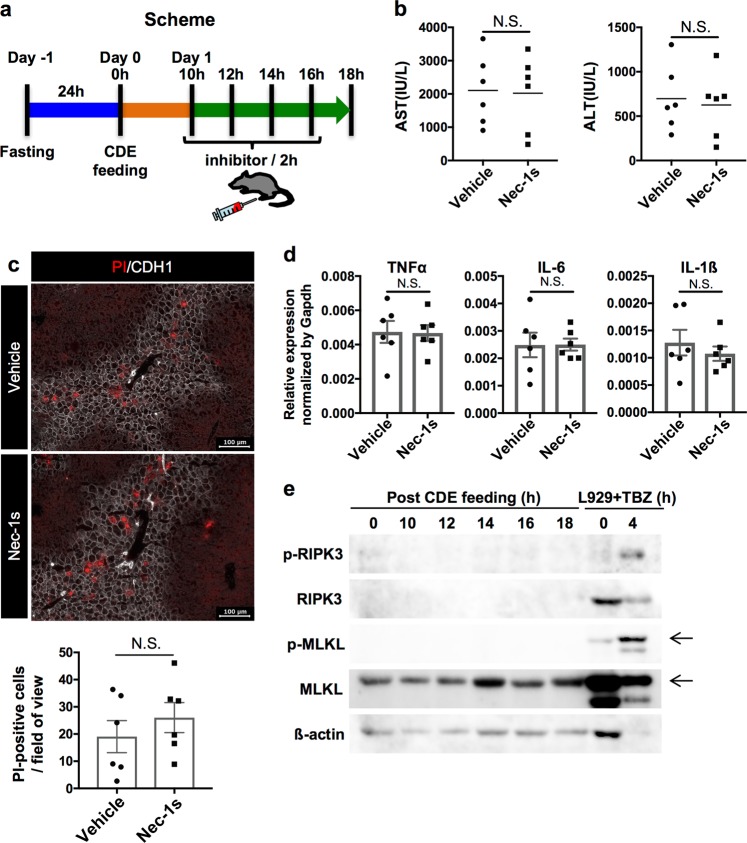
Evaluation of the effect of necroptosis inhibition in the CDE diet model. **a** Experimental scheme for the administration of inhibitory agent. **b** Serum liver injury marker levels of vehicle or Nec-1s (5 mg/kg) treated mice after 18 h of the CDE diet feeding (*n* = 6). NS not significant. **c** Detection of necrotic cells in the CDE-fed mouse treated with vehicle or Nec-1s. Scale bar = 100 µm. The number of PI-positive cells was evaluated in 10 non-overlapping fields of view for each biological sample. The data are shown as the means ± SEM (*n* = 6). NS not significant. **d** Gene expression analysis of inflammatory cytokines by quantitative RT-PCR. All data are normalized to Gapdh and shown as the means ± SEM (*n* = 6). NS not significant. **e** Western blot analysis of phosphorylated RIPK3 (p-RIPK3), RIPK3, phosphorylated MLKL (p-MLKL), MLKL, and β-actin in the liver extracts after the CDE diet feeding. L929 cells treated with TNFα, BV, and ZVAD-FMK (L929 + TBZ) were used as control for necroptotic cell death

Since necrosis is likely to be a trigger of inflammation, we further investigated the type of necrosis occurring at the initiation of steatohepatitis. Firstly, we examined whether “necroptosis” was involved in the initial necrosis of this model, because necroptosis has been implicated in inflammation and fibrosis in MCD diet or high-fat diet models^5,20^. To address this issue, we administered a chemical inhibitor of necroptosis, Necrostatin-1s (Nec-1s), in four doses at 10, 12, 14, and 16 h post CDE feeding, and then evaluated the liver at 18 h by measurement of serum AST, ALT, in vivo necrosis assay, and gene expression analysis of inflammatory cytokines (Fig. 4a). We chose to begin administration of Nec-1s at 10 h as we had observed no sign of necrosis at this time point within our experimental settings, based on normal levels of serum AST and ALT (Fig. 3d). We hypothesized that if the initial necrosis was necroptosis, the symptoms of steatohepatitis should be successfully suppressed by Nec-1s administration. However, since the administration of Nec-1s failed to reduce serum AST and ALT (Fig. 4b), necroptosis is not likely to be the initial cell death in this model. Consistently, there was no apparent difference in the emergence of PI-positive necrotic cells and gene expression of inflammatory cytokines between control and Nec-1s-treated mice (Fig. 4c, d), suggesting that Nec-1s could not block the initial necrosis. Next, we investigated the phosphorylation of two key regulators of necroptosis, RIPK3 and MLKL^21^, by western blot analysis. As a result, neither the phosphorylation of RIPK3 nor MLKL was detected in the liver samples from 10 to 18 h post feeding (Fig. 4e).

**Fig. 5:**
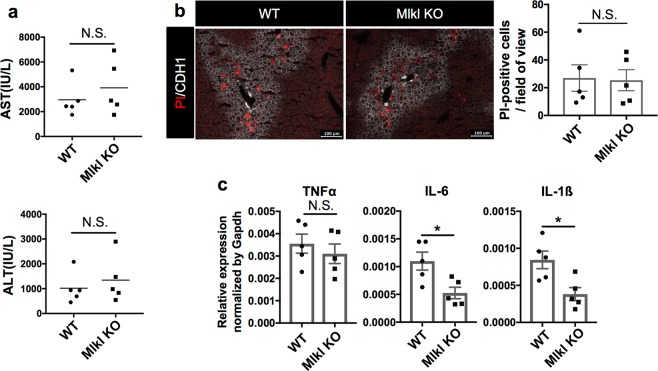
Evaluation of necrotic cell and inflammation in the liver of CDE-fed WT and Mlkl KO mice. **a** Serum liver injury marker levels of WT and Mlkl KO mice after 18 h of the CDE diet feeding (*n* = 5). NS not significant. **b** Detection of necrotic cells in the liver of WT and Mlkl KO mice at 18 h post CDE feeding by the in vivo necrosis assay. The number of PI-positive cells was evaluated in 10 non-overlapping fields of view for each biological sample. The data are shown as the means ± SEM (*n* = 5). NS not significant. **c** Gene expression analysis of inflammatory cytokines by quantitative RT-PCR. All data are normalized to Gapdh and shown as the means ± SEM (*n* = 5; *P *<* 0.05)

To further confirm that necroptosis is unlikely to be responsible to the initial cell death in the CDE model, knockout (KO) mice lacking in Mlkl, a terminal executor of necroptosis, were fed with a CDE diet at 0 h, and then sacrificed at 18 h post feeding for further analyses. The measurement of serum injury markers and PI staining revealed that hepatic necrosis occurred in Mlkl KO as well as WT mice similarly, strongly suggesting that necroptosis is not likely to be the initial cell death in this model (Fig. 5a, b). However, Mlkl KO mice showed a significant down-regulation of gene expression in some inflammatory cytokines (Fig. 5c), suggesting that necroptosis of non-hepatocyte (e.g. immune cells) may be involved in the exacerbation of inflammation at the later stage of steatohepatitis.

### Ferroptosis is a promising candidate for the initiating cell death in steatohepatitis

**Fig. 6:**
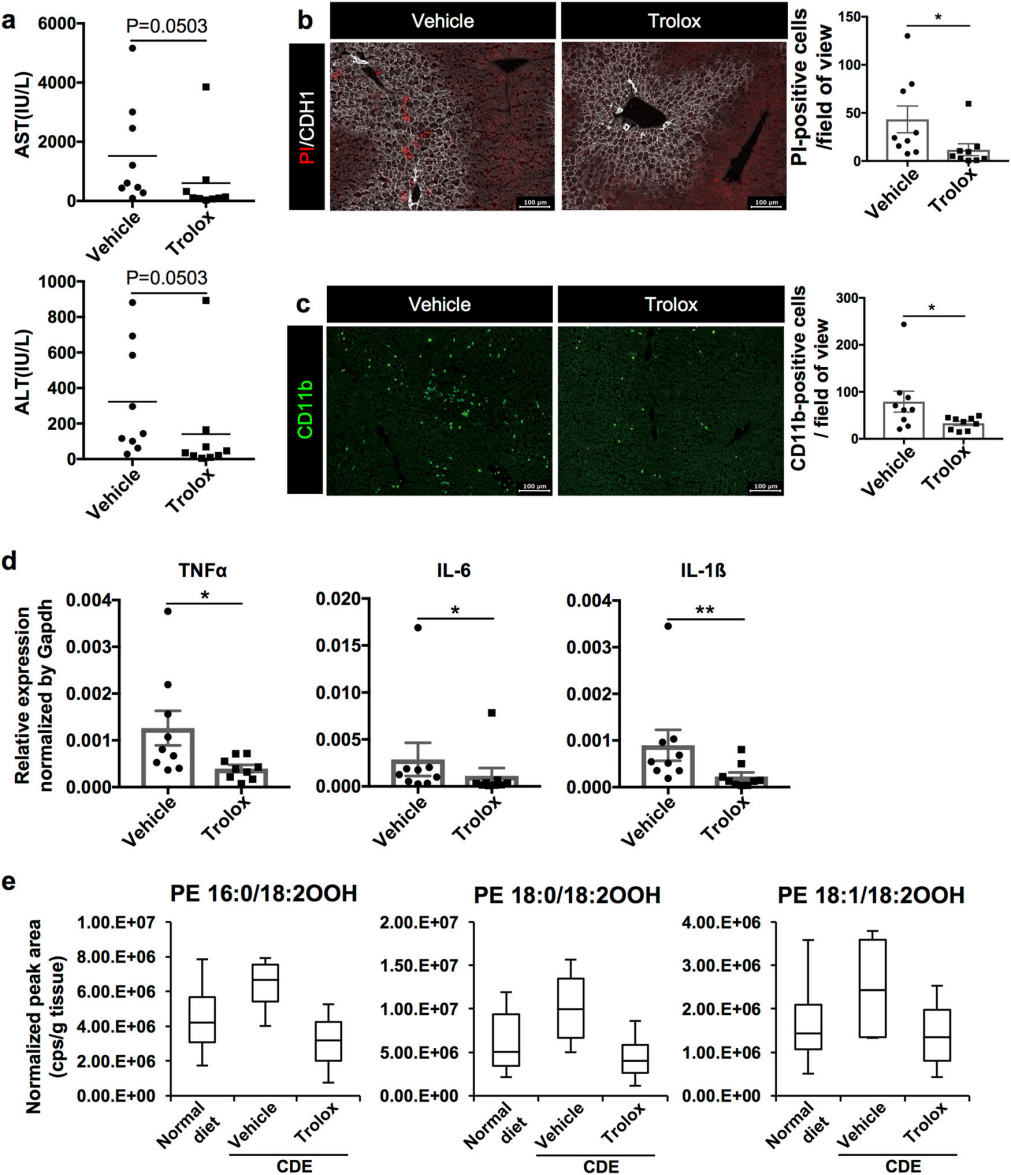
Evaluation of the effect of ferroptosis inhibitor, Trolox, in the CDE diet model. **a** Serum liver injury marker levels in control (vehicle) or Trolox (100 mg/kg) treated mice (*n* = 9). **b** Detection of necrosis in the CDE-fed mice treated with vehicle or Trolox by the in vivo necrosis assay. The number of PI-positive cells was evaluated in 10 non-overlapping fields of view for each biological sample. The data are shown as the means ± SEM (*n* = 9; *P *<* 0.05). **c** Immunohistochemical analysis of CDE-fed mouse with vehicle or Trolox for CD11b. The number of CD11b-positive cells was evaluated in 10 non-overlapping fields of view for each mouse. The data are shown as the means ± SEM (*n* = 9; *P *<* 0.05). **d** Gene expression analysis of inflammatory cytokines by quantitative RT-PCR (*n* = 9). All data are normalized to Gapdh and shown as the means ± SEM (*n* = 9; *P *<* 0.05, **P *<* 0.01). e Measurement of oxidized PEs in the liver. The level of each oxidized PE in the liver sample was compared among normal diet-fed mice, CDE diet-fed mice, and Trolox-treated CDE diet-fed mice (*n* = 4). Scale bar = 100 µm

The accumulation of iron in the liver is considered as an aggravating factor of NASH^22,23^. Therefore, we have focused on ferroptosis as a possible initiator of steatohepatitis. Similar to the administration of Nec-1s into the CDE-fed mice, the effect of rosiglitazone (ROSI), an inhibitor of ACSL4, and Trolox, an antioxidant vitamin E analog, was examined, respectively. Both agents have been reported to inhibit ferroptosis previously^10,13^. The administration of ROSI showed a tendency towards amelioration of hepatic cell death judging from serum injury markers at 18 h post CDE feeding (Supplementary Figure S4). On the other hand, the administration of Trolox dramatically decreased the level of serum liver damage markers and the number of PI-positive necrotic cells even at 18 h post feeding (Fig. 6a, b). Also, gene expression of inflammatory cytokines and infiltration of inflammatory cells was significantly suppressed (Fig. 6c, d). Next, we compared the amount of phospholipid peroxides in the liver between control and Trolox-treated mice, because oxygenated PE has been recently reported to be involved in the pathway for ferroptotic signaling^12,13^. As a result, the amount of oxygenated PE was increased in the liver of CDE diet-fed mice compared with that of normal chow-fed mice. As expected, together with the amelioration of liver damage, the elevated amount of oxygenated PE in the livers of CDE-fed mice was ameliorated by the administration of Trolox (Fig. 6e). These results strongly suggested that ferroptosis is implicated in the pathogenesis of NASH.

### Effect of iron chelating agents on the suppression of initial necrosis in steatohepatitis

**Fig. 7:**
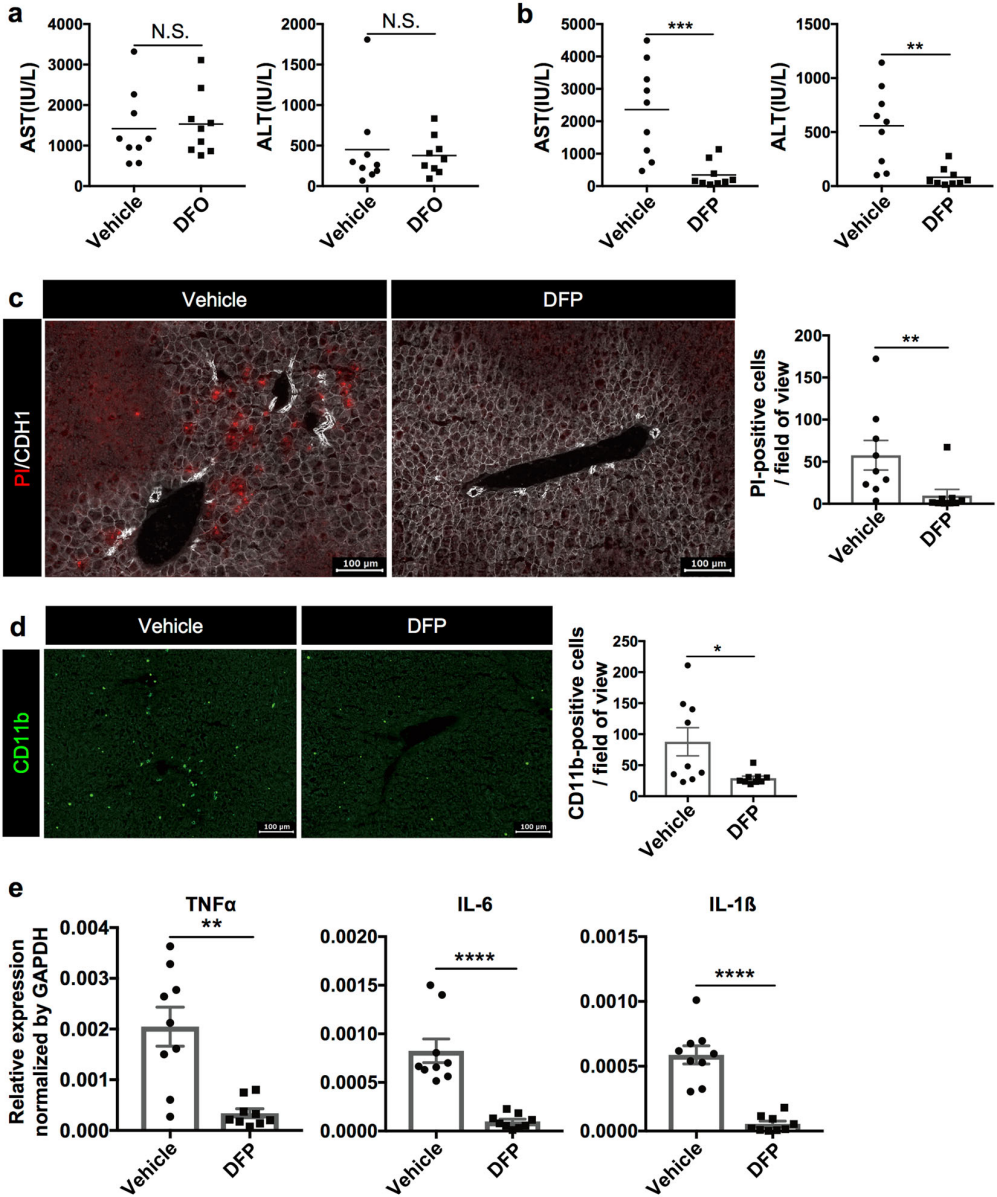
Evaluation of the effect of iron chelators in the CDE diet model. **a** Serum liver injury marker levels in the CDE-fed mice treated with vehicle or DFO (100 mg/kg) (*n* = 9). NS not significant. The serum data are shown as dot plots and mean. **b** Serum liver injury marker levels of the CDE-fed mice treated with vehicle or DFP (100 mg/kg) (*n* = 9; **P *<* 0.01, ***P < 0.001). **c** Detection of necrotic cells in the CDE-fed mice treated with vehicle or DFP. The number of PI-positive cells was evaluated in 10 non-overlapping fields of view for each biological sample. The data are shown as the means ± SEM (*n* = 9; **P *<* 0.01). **d** Immunohistochemical analysis of CDE-fed mouse treated with vehicle or DFP for CD11b. The number of CD11b-positive cells was evaluated in 10 non-overlapping fields of view for each biological sample. The data are shown as the mean ± SEM (*n* = 9; *P < 0.05). **e** Gene expression analysis of inflammatory cytokines by quantitative RT-PCR. The gene expression data are normalized to Gapdh and shown as the means ± SEM (*n* = 9; **P *<* 0.01, ****P *<* 0.0001). Scale bar = 100 µm

To confirm whether ferroptosis is the trigger for steatohepatitis, we examined the effect of iron chelating agents, which are another type of ferroptosis inhibitor. Iron is known to play a catalytic role for the fenton reaction, which promotes the production of lipid peroxides required for ferroptosis. Because Deferoxamine (DFO) is commonly used in the study of ferroptosis, we administered DFO to the CDE-fed mice. Contrary to our expectation, the administration of DFO did not ameliorate the increase of serum liver injury markers (Fig. 7a), suggesting that the initial hepatic necrosis was not suppressed by DFO treatment. Interestingly, however, the administration of another iron chelator Deferiprone (DFP), which has higher membrane permeability than DFO^24^, markedly suppressed the elevation of serum liver injury markers and the emergence of PI-positive necrotic cells almost completely (Fig. 7b, c). As was the case with Trolox, infiltration of immune cells and inflammatory cytokine level were also suppressed by DFP administration (Fig. 7d, e), suggesting that intracellular iron plays a crucial role in necrotic cell death rather than extracellular one in the liver. Taken together, these data strongly suggested that ferroptosis is the initiating cell death that induces inflammation at the onset of steatohepatitis.

## Discussion

Cell death is a commonly observed event in most human diseases. While apoptosis is a regulated cell death accompanied by intracellular signaling cascades^25^, necrosis had been considered as unregulated or accidental cell death. However, the discovery of a regulated form of necrotic cell death, namely “programmed necrosis” brought about a change in our view of necrosis, and much effort has been made to elucidate the molecular mechanisms underlying the execution of necrotic cell death^10,26,27^. Along with the advance in understanding of each programmed cell death, much attention has been paid to the pathophysiological roles of multiple types of cell death in a wide range of diseases including hepatic failure. Unlike the detection method of apoptosis such as TUNEL or CC3 staining, the difficulty of identifying necrosis in vivo has hampered the acquisition of regional information about apoptosis and necrosis in a wide area of injured tissue. In this study, we designed a new strategy by applying the PI staining for in vivo analysis and showed that initial cell death occurred predominantly in the periportal area at the onset of steatohepatitis. This method may be applicable to the other tissues, organs, or disease models.

In the pathogenesis of NASH, there is accumulating evidence for the involvement of apoptosis in disease progression. The cytokeratin-18 fragment, which is a molecule cleaved by caspase and released from apoptotic hepatocytes, is used as one of the diagnostic biomarkers in NASH patients^28,29^. Although apoptosis inhibition is known to suppress fibrosis in murine NASH models, serum ALT levels are less affected, suggesting that the other types of cell death are involved in the pathogenesis of NASH^30^. In fact, necroptosis has been recently reported to contribute to the progression of NASH. RIPK3 and MLKL, which are key regulators of necroptosis, were upregulated in both murine NASH models and NASH patients^20^, and necroptosis inhibition in Ripk3 KO mice shows decreased serum AST and ALT, inflammatory cytokines, and fibrosis^5^. More recently, pyroptosis, another type of programmed necrosis, has also been reported to play a role in the pathogenesis of NASH in Gasdermin D KO mouse^31^. These studies strongly suggest that the pathological progression of NASH is governed by multiple types of cell death. However, their involvement in the initial hepatic cell death at the onset of steatohepatitis has remained unclear because it is difficult to specify when hepatocytes begin to die in fatty liver in long-term experimental settings.

On the other hand, ferroptosis is a recently identified programmed necrosis, which is an iron- and lipid hydroperoxide-dependent nonapoptotic cell death. For the pathogenesis of NASH, oxidative stress induced by the abnormal accumulation of lipid is considered as an important initiation factor^32^. There is further evidence to suggest the involvement of ferroptosis in NASH patients as follows: secondary products of lipid peroxidation such as malondialdehyde and 4-hydroxinonenal are utilized as oxidative stress markers in NASH patients^33,34^. Vitamin E, which is an antioxidant that suppresses peroxidizing lipids, reduces serum ALT in NASH patients^35^. Moreover, the iron accumulation resulting from metabolic aberration is also believed to be an aggravating factor because of the following reasons: liver siderosis is observed in some of NASH patients^22^. The iron removal by phlebotomy is known to ameliorate liver damage and serum ALT^36^. NASH is exacerbated in primary hemochromatosis patients^37^. It is therefore plausible that ferroptosis is implicated in the pathogenesis of NASH. Nonetheless, there are few papers demonstrating the implication of ferroptosis in NASH. The reason why the possible role of ferroptosis has not been reported in murine model of NASH is as follows: the exact execution factors and signaling cascades of ferroptosis remain elusive, while those of apoptosis, necroptosis, and pyroptosis are well documented. Therefore, KO mice lacking in important components such as caspase family members, Ripk3, Mlkl, and Gsdmd, are useful for evaluating the contribution of the above three types of cell deaths to the pathogenesis of NASH^5,30,31^. In contrast, methods of ferroptosis inhibition in vivo are currently restricted to the use of several inhibitory agents such as antioxidants and iron chelators. Conventionally, in previous experiments using KO mice, the symptoms such as inflammation, fibrosis, and carcinogenesis have been examined after long-term feeding of MCD diet or high-fat diet^38,39^. Considering that the liver is the major organ of xenobiotic metabolism, continuous administration of chemical agents may not be suitable for follow-up examination in chronic liver injury models. Given that multiple types of cell death are involved in the progression of disease state in NASH, the effect of ferroptosis inhibition after the onset of steatohepatitis may become limited in conventional NASH models. Therefore, taking advantage of the CDE diet model, in which the hepatic cell death occurs within a short period of time in the context of steatosis, we focused on the initial cell death at the onset of steatohepatitis.

In this study, we demonstrated that necrosis precedes apoptosis at the early stage of steatohepatitis and that two ferroptosis inhibitors, Trolox and DFP, suppressed necrotic cell death, infiltration of inflammatory cells, and inflammatory cytokine expression almost completely. In addition, we showed that the amount of oxygenated PE, which is implicated in the ferroptosis pathway, was increased in the liver of the CDE-fed mice compared with normal diet-fed mice, and that the increase of oxygenated PE was lowered to a normal liver level by Trolox treatment. Consistently, it is reported that the hepatic PC/PE ratios are decreased in NASH patients^40^. These results suggested that ferroptosis is the initial necrosis to be the trigger for steatohepatitis.

By contrast, necroptosis inhibition by using Nec-1s or Mlkl KO mice could not block the initial necrotic cell death. However, Mlkl KO mice showed a tendency of decreasing inflammatory cytokine expression, implying that necroptosis in the non-parenchymal cells might be involved in the exacerbation of inflammation in NASH. Although it is unclear whether ferroptosis is followed by necroptosis and apoptosis in the progression stage of NASH, there is evidence supporting the hypothesis that ferroptosis occurs in parallel with the other types of cell death in NASH patients as described above. Total inhibition of multiple cell deaths relevant to NASH pathogenesis could prove to be effective in the treatment of NASH. Therefore, elucidation of the correlation among multiple types of cell death in the pathological process of NASH is crucial.

In conclusion, our data suggested that ferroptosis plays a potential role as the trigger for steatohepatitis, leading to liver damage, infiltration of immune cells, and inflammatory reaction. Ferroptosis is a promising therapeutic target for the prevention of onset of steatohepatitis. Therefore, it is necessary to take ferroptosis into account for the therapeutic strategy of NASH. Our findings will provide a new insight into the type of cell death relevant to the pathogenesis of NASH.

## Materials and methods

### Mice and models of steatohepatitis by the CDE diet and CDAA-based high-fat diet

Male C57BL/6J mice were purchased from CLEA Japan, Inc. *Mlkl−/−* mice were provided by M. Pasparakis and described prebiously^41^. All animal experiments were conducted in accordance with institutional procedures and admitted by the Animal Care and Use committee of the Institute for Quantitative Biosciences, the University of Tokyo and Research Institute, the National Center for Global Health and Medicine. We used 5- to 6-week-old mice for feeding CDE diet (MP Biomedicals, USA) and CDAA-based high-fat diet (CDAHFD, A06071302, Research diet, USA), and harvested liver and blood samples for further analyses.

### Measurement of serum ALT and AST

The serum liver injury markers, ALT and AST were measured using Transaminase C-II Test Wako (Wako, Japan) according to the manufacturer’s instruction.

### In vivo necrosis assay by PI injection

PI (Sigma, USA) was dissolved in phosphate-buffered saline (PBS) at a final concentration of 25 µg/ml. For the detection of necrotic cells in the liver, the PI solution was injected into mice intravenously via the tail vein. The liver samples were harvested and snap frozen in liquid nitrogen 10 min later. The frozen block was cut into slices with 8 µm thickness by using Microtome Cryostat HM 525 (Thermo Fisher Scientific Industries, Osaka, Japan) and mounted on slide glasses. After imaging of PI-stained tissues, the samples were counterstained with Hoechst, anti-Cleaved caspase-3 (CC3, #9446, CST, USA, 1:300), anti-Cadherin1 (CDH1, #3195, CST, USA, 1:200) and anti-CK19 (DSHB, USA, 1:1000) antibodies. Merged images were captured using BZ-X710:BZ-X Viewer (KEYENCE, Japan). The number of PI-positive cells was counted using Hybrid Cell Count function in the Dual Signal Extraction mode of BZ-X Analyzer. An average value of 10 random images per mouse was treated as a representative value for the mouse.

### CCl_4_ liver injury model

We injected CCl_4_ dissolved in olive oil (2 mg/kg; Wako) into 8-week-old mice intraperitoneally and harvested liver samples after 24 h. Prior to the in vivo PI staining, we collected blood samples for measurement of serum AST and ALT.

### Inhibition of cell death in the CDE-fed liver by using inhibitors

We used 5- to 6-week-old mice for this experiment. After fasting for 24 h, the CDE diet was fed at 0 h. The inhibitor was injected intraperitoneally in four doses every 2 h from 10 to 16 h and then mice were sacrificed for evaluation at 18 h. After the blood samples were collected, the in vivo necrosis assay was performed as described before. The final dose of used inhibitor used is as follows: Necrostatin-1s (5 mg/kg; Focus Biomolecules, USA), Rosiglitazone (5 mg/kg; Sigma), Trolox (100 mg/kg; Sigma), Deferiprone (100 mg/kg; Sigma), and Deferoxamine (100 mg/kg; Sigma). For injection, Trolox was initially dissolved in a small amount of DMSO (Sigma), and then diluted in olive oil. Nec-1s and Rosiglitazone were first dissolved in DMSO, and then diluted in PBS. All other inhibitors were diluted in PBS. Similarly, wild-type (WT) and *Mlkl* KO mice were analyzed as described above.

### HE, Picro-Sirius Red, and Oil Red O staining

Frozen sections of liver sample with 8 µm thickness were fixed with 4% paraformaldehyde at room temperature for HE staining. For evaluation of liver fibrosis, Picro-Sirius Red staining was performed as described before^42^. For Oil Red O staining, the sections were stained with Mayer’s hematoxylin solution (Wako) after rinsing with PBS. Then, the sections were washed with running water, incubated in 60% isopropanol (Wako), stained with Oil Red O staining solution (SIGMA), and reincubated in 60% isopropanol. Finally, the sections were washed with running water and mounted with fluoromount (Cosmo Bio, Japan).

### Immunohistochemistry

For immunohistochemistry, fixed samples with 4% paraformaldehyde were blocked with PBS containing 1% bovine serum albumin and 0.3% Triton X-100, and incubated with anti-CC3, anti-CK19, and anti-CD11b (BD Pharmingen, USA; 1:200) antibodies at 4 °C overnight. The second antibodies conjugated with Alexa Fluor 488 or Alexa Fluor 647 (Thermo Fisher Scientific, USA, 1:800) were used for immunofluorescence. All images of tissue sections were captured using BZ-X710:BZ-X Viewer (Keyence, Japan).

### Total RNA extraction and quantitative RT-PCR

Total RNA was extracted from liver tissues with TRIzol reagent (Invitrogen, Carlsbad, CA, USA). The cDNA was synthesized from RNA with PrimeScript^TM^ RT Master Mix (Takara, Japan) for quantitative RT-PCR. The expression level normalized to Gapdh was analyzed by using the LightCycler 480 (Roche, Basel, Switzerland). The primer sequences are listed in Table 1.

**Table 1:** Primer sequences for quantitative RT-PCR

Genes	Forward primer sequence (5′ → 3′)	Reverse primer sequence (5′ → 3′)
TNFα	TCTTCTCATTCCTGCTTGTGG	GGTCTGGGCCATAGAACTGA
IL-6	GCTACCAAACTGGATATAATCAGGA	CCAGGTAGCTATGGTACTCCAGAA
IL-1ß	AGTTGACGGACCCCAAAAG	AGCTGGATGCTCTCATCAGG
TGFß	TGGAGCAACATGTGGAACTC	CAGCAGCCGGTTACCAAG
Collagen 1a1	CATGTTCAGCTTTGTGGACCT	GCAGCTGACTTCAGGGATGT
Collagen 3a1	TCCCCTGGAATCTGTGAATC	TGAGTCGAATTGGGGAGAAT

### Western blot analysis

Proteins were extracted with RIPA lysis buffer from liver tissues and each concentration was measured by Bradford protein assay. Protein samples were electrophoresed on polyacrylamide gels and transferred onto PVDF membranes. Membranes were blocked with 5% skim milk in PBS and incubated with anti-MLKL Rat Ab (#MABC604; EMD Millipore, USA, 1:1000), anti-Phospho-MLKL (Ser345) Rabbit Ab (Mouse specific) (#62233; CST, USA, 1 : 1000), anti-Phospho-RIP3 (Thr231/Ser232) rabbit antibody (Mouse specific) (#57220; CST, USA, 1:1000), anti-CC3 (#9446; CST, USA, 1:500), and anti-ß-actin (#4967; CST, USA, 1:1000; 622101 Biolegend, Japan, 1:1000)) at 4 °C overnight. After washing with TBST, membranes were incubated with secondary antibody at room temperature for 60 min and washed with TBST. The immunoblot was then imaged according to the manufacturer’s instructions from Clarity^TM^ Western ECL Substrate (Bio Rad, USA), and semi-quantitatively measured by Multi Gauge (Fuji Film, Japan). For the preparation of necroptotic sample, L929 cells were cultured in the presence of 20 μM ZVAD-FMK for 30 min, followed by the addition of 20 ng/ml of mouse TNFα and 1 μM BV. After 4 h of culture, the cell lysate was recovered for western blotting.

### Sample preparation for LC-MS

For lipid extraction, the liver of mice (0.1 g) were homogenized with 1 ml methanol including an internal standard (17:0-lysoPC) using a glass homogenizer and extracted after stand for 1 h at on ice. The samples were centrifuged (7000 r.p.m., 4 °C, 5 min) to remove cellular and protein materials, then the supernatant was diluted with 10 volumes of water and then adjusted to pH 3.0 with 0.1 N HCl. The samples were applied to preconditioned (20 ml of methanol and 20 ml of water) C18 Sep-Pak cartridges (500 mg; Waters, Millford, MA, USA), and washed with 20 ml of water to exclude nonvolatile ions followed by 10 ml of hexane to exclude cholesterols and neutral lipids. The samples were eluted with 10 ml of methanol to obtain oxidized phospholipids. The lipid extracts were dried under a gentle stream of nitrogen, dissolved in 1 ml methanol, and stored at −80 °C until use.

### LC-ESI-MS/MS system

The LC-ESI-MS/MS analysis was carried out using a QTRAP 4500 quadropole linear ion trap hybrid mass spectrometer (AB Sciex, Concord, ON, Canada) with a Nexera XR high-performance liquid chromatography (Shimadzu Co., Kyoto, Japan). The sample was subjected to LC-ESI-MS/MS analysis using the XBridge BEH C18 column (Waters). Sample was injected by the autosampler, and the phospholipid fractions were separated by a step gradient with mobile phase A (acetonitrile/methanol/water = 2:2:1 v/v/v containing 0.1% formic acid and 0.028% ammonia): mobile phase B (isopropanol containing 0.1% formic acid and 0.028% ammonia) ratios of 100:0 (0–5 min), 50:50 (5–25 min), 50:50 (25–59 min), 100:0 (59–60 min), and 100:0 (60–75 min) at a flow rate of 70 μL/min and a column temperature of 30 °C. The multiple reaction monitoring was carried out to detect specific oxidized phospholipids. MS/MS analysis was carried out in negative ion mode with the following settings, ion spray voltage, −4500 V; curtain gas (N2), 30 arbitrary units; collision gas (N2), “medium”; declustering potential, −60 to −170 V, collision energy, −40 to −44 eV; temperature, 500 °C. For the detection of PEOOH, deprotonated ions ([M-H]^−^) were selected as a precursor ion, and the peroxidized fatty acyl chain were selected as product ions ([M-H-H_2_O]^−^).

### Statistical analyses

Statistical analyses were performed using GraphPad Prism software. Statistical significance between two groups was evaluated using a two-tailed Mann–Whitney *U*-test. For comparison of more than three groups, Kruskal–Wallis test was applied. *P* < 0.05 was considered statistically significant. The exact number of biological samples was described in each figure legend. There was no exclusion of outliers in all experiments. Group allocation was performed without any bias.

## Supplementary information

Supplementary Fig.S1.

Supplementary Fig.S2.

Supplementary Fig.S3.

Supplementary Fig.S4.
