# Supplementary figures and images for: Hepatic ferroptosis plays an important role as the trigger for initiating inflammation in nonalcoholic steatohepatitis
Source: Cell Death Dis. 2019 Jun 18;10(6):449. doi: 10.1038/s41419-019-1678-y (PMC6579767; doi:10.1038/s41419-019-1678-y)

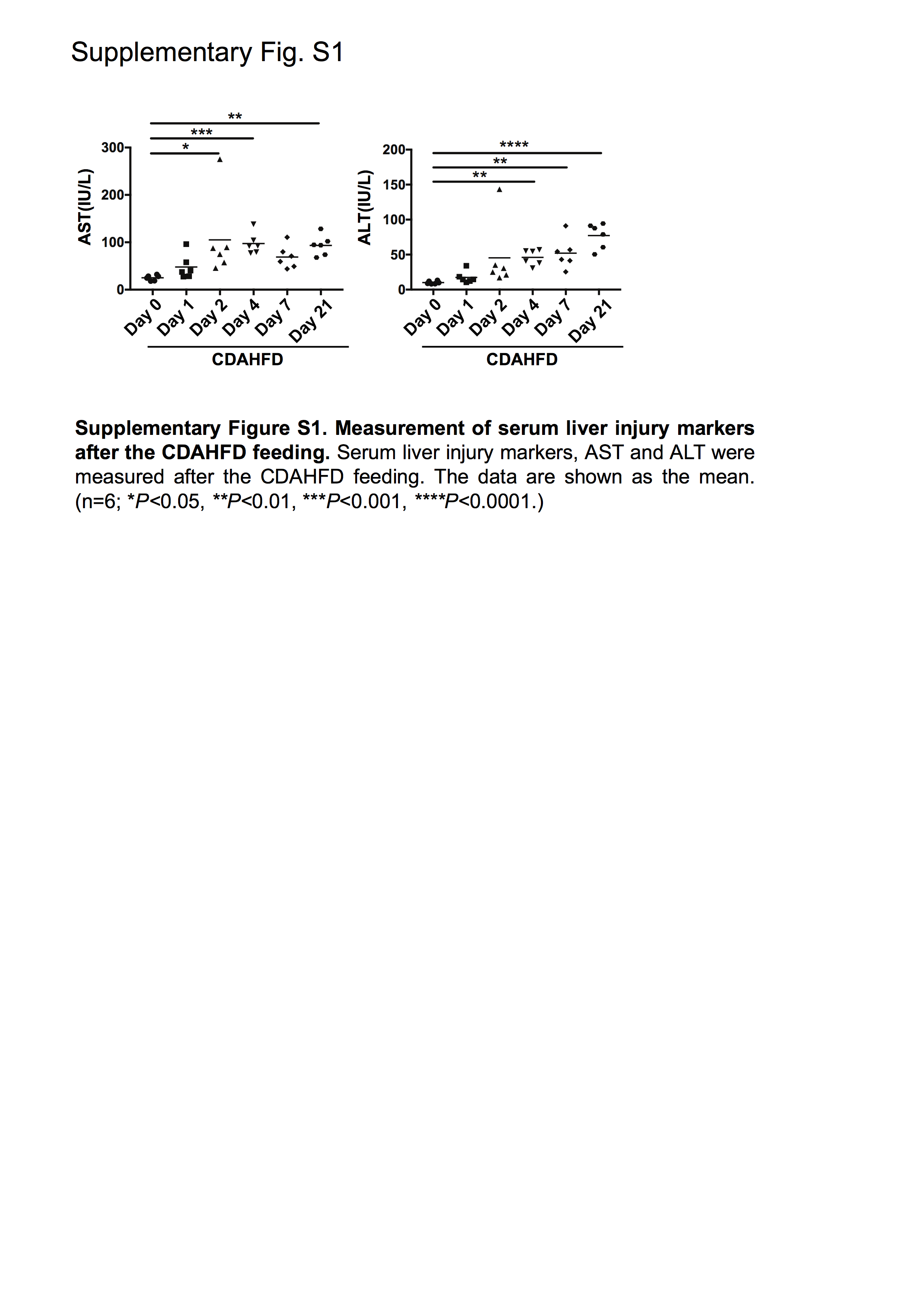

Supplement: Supplementary file 1 — Supplementary Fig.S1. [file 41419_2019_1678_MOESM1_ESM.tif]

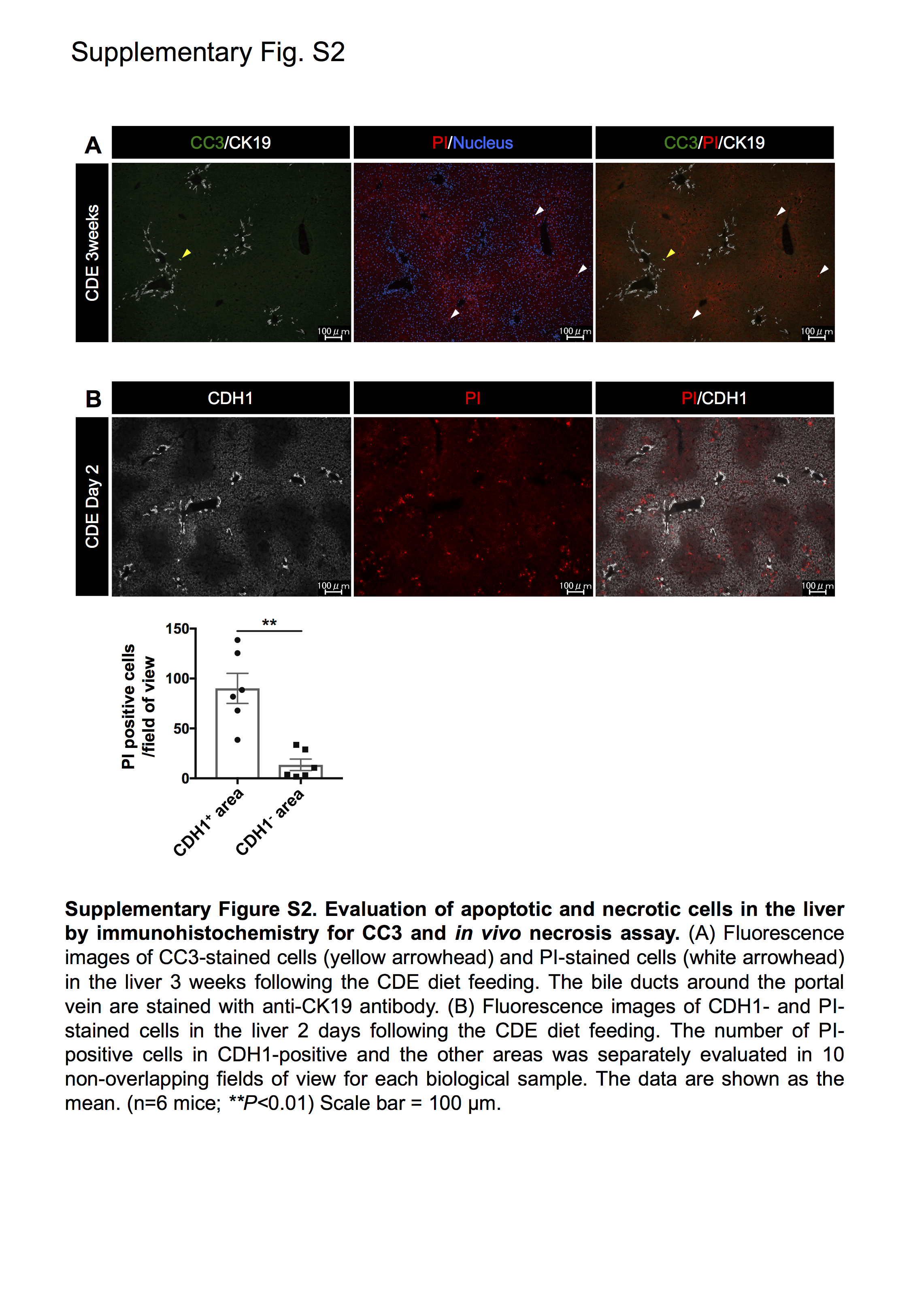

Supplement: Supplementary file 2 — Supplementary Fig.S2. [file 41419_2019_1678_MOESM2_ESM.tif]

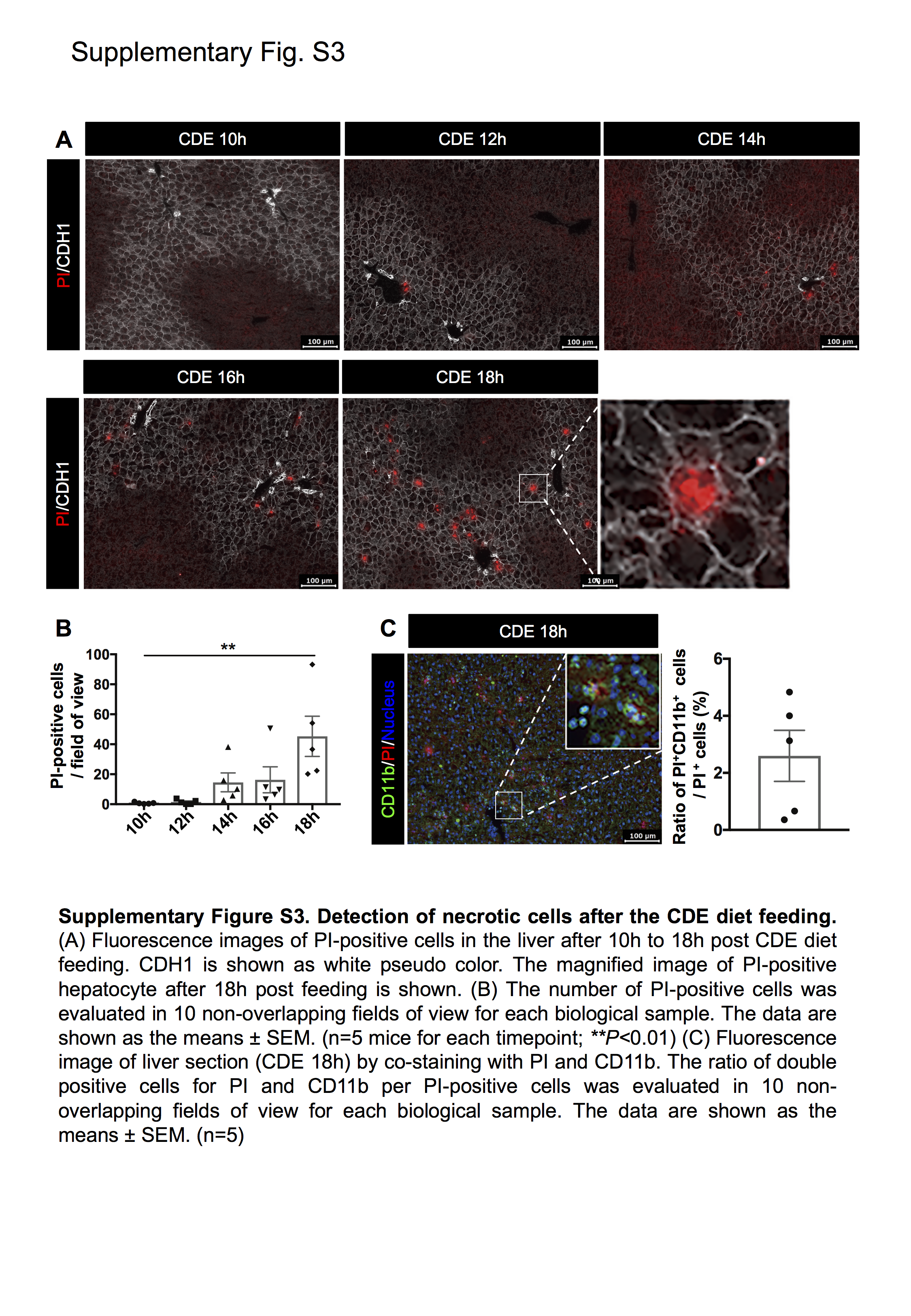

Supplement: Supplementary file 3 — Supplementary Fig.S3. [file 41419_2019_1678_MOESM3_ESM.tif]

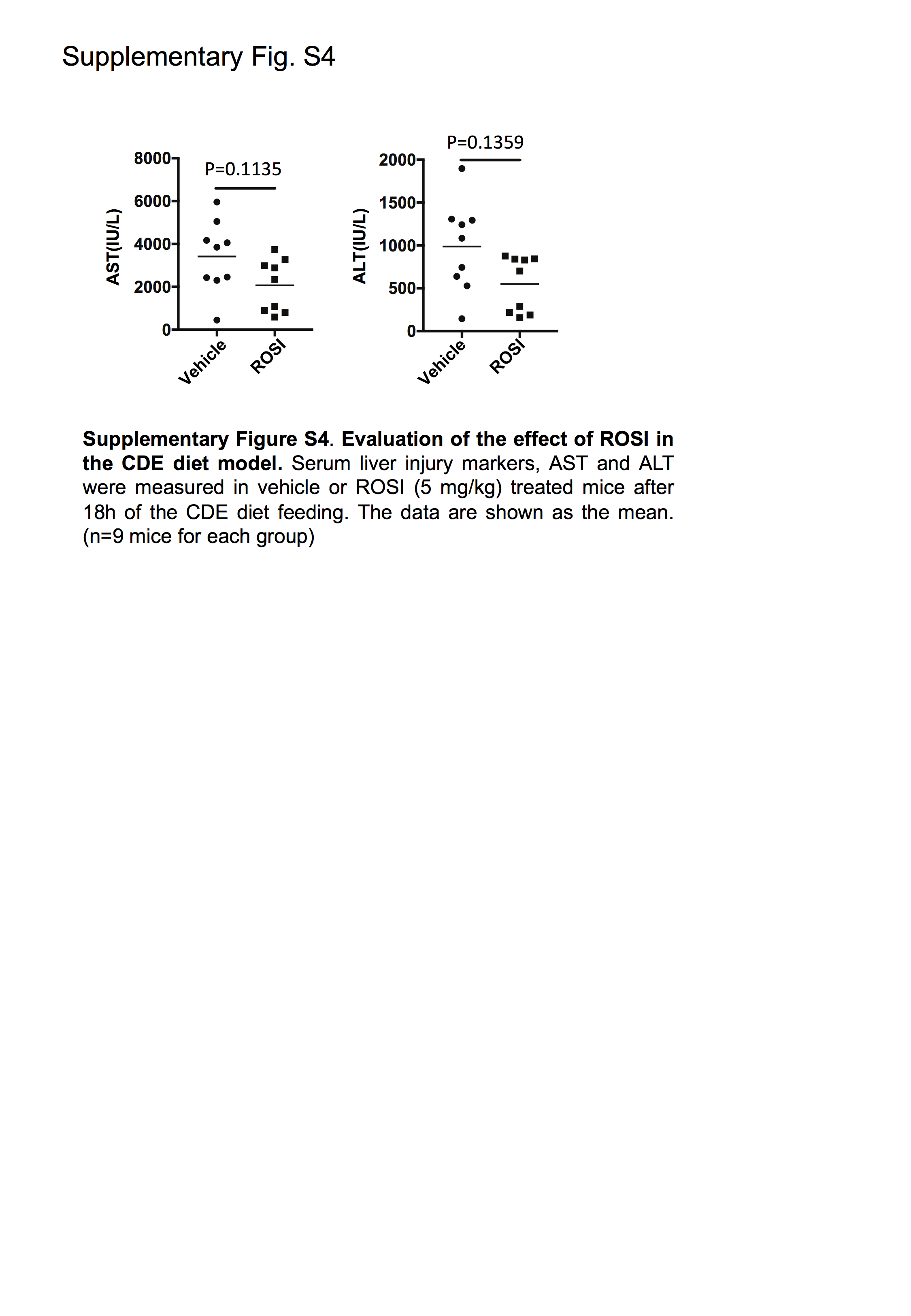

Supplement: Supplementary file 4 — Supplementary Fig.S4. [file 41419_2019_1678_MOESM4_ESM.tif]
